# KG4SL: knowledge graph neural network for synthetic lethality prediction in human cancers

**DOI:** 10.1093/bioinformatics/btab271

**Published:** 2021-07-12

**Authors:** Shike Wang, Fan Xu, Yunyang Li, Jie Wang, Ke Zhang, Yong Liu, Min Wu, Jie Zheng

**Affiliations:** School of Information Science and Technology, ShanghaiTech University, Shanghai 201210, China; School of Information Science and Technology, ShanghaiTech University, Shanghai 201210, China; School of Life Science and Technology, ShanghaiTech University, Shanghai 201210, China; School of Information Science and Technology, ShanghaiTech University, Shanghai 201210, China; School of Information Science and Technology, ShanghaiTech University, Shanghai 201210, China; Shanghai Institute of Microsystem and Information Technology, Chinese Academy of Sciences, Shanghai 200050, China; Joint NTU-UBC Research Centre of Excellence in Active Living for the Elderly, Nanyang Technological University, Singapore 639798, Singapore; Institute for Infocomm Research, Agency for Science, Technology and Research (A*STAR), Singapore 138632, Singapore; School of Information Science and Technology, ShanghaiTech University, Shanghai 201210, China; Shanghai Engineering Research Center of Intelligent Vision and Imaging, Shanghai, 201210, China

## Abstract

**Motivation:**

Synthetic lethality (SL) is a promising gold mine for the discovery of anti-cancer drug targets. Wet-lab screening of SL pairs is afflicted with high cost, batch-effect, and off-target problems. Current computational methods for SL prediction include gene knock-out simulation, knowledge-based data mining and machine learning methods. Most of the existing methods tend to assume that SL pairs are independent of each other, without taking into account the shared biological mechanisms underlying the SL pairs. Although several methods have incorporated genomic and proteomic data to aid SL prediction, these methods involve manual feature engineering that heavily relies on domain knowledge.

**Results:**

Here, we propose a novel graph neural network (GNN)-based model, named KG4SL, by incorporating knowledge graph (KG) message-passing into SL prediction. The KG was constructed using 11 kinds of entities including genes, compounds, diseases, biological processes and 24 kinds of relationships that could be pertinent to SL. The integration of KG can help harness the independence issue and circumvent manual feature engineering by conducting message-passing on the KG. Our model outperformed all the state-of-the-art baselines in area under the curve, area under precision-recall curve and F1. Extensive experiments, including the comparison of our model with an unsupervised TransE model, a vanilla graph convolutional network model, and their combination, demonstrated the significant impact of incorporating KG into GNN for SL prediction.

**Availability and implementation:**

: KG4SL is freely available at https://github.com/JieZheng-ShanghaiTech/KG4SL.

**Supplementary information:**

Supplementary data are available at *Bioinformatics* online.

## 1 Introduction

Complex biological systems cannot be composed by a large number of genes acting independently, but rely on the interactions between genes which can be further classified into enhancing and suppressive effects (Dhabhar, 2009). The suppressive effects characterize the situation that, when mutations occur simultaneously in a pair of genes, some important functions will be deactivated which seriously decrease cell viability, whereas the mutation in a single gene might not affect the cell viability. A common type of suppressive effect is synthetic lethality (SL; Dobzhansky, 1946), which has been a promising strategy for cancer medicine (Ashworth *et al.*, 2011). If a specific gene is found to be inactivated in tumor cells, drugs that suppress its SL partner gene can cause tumor cells to die but spare normal cells (Hartwell *et al.*, 1997). Hence, SL is a gold mine of anti-cancer drug targets, and intensive efforts have been exerted to identify SL gene pairs. High-throughput wet-lab screening methods, including chemical liraries (Simons *et al.*, 2001), pooled RNAi screening (Luo *et al.*, 2009) and CRISPR-based genome editing technology (Du *et al.*, 2017) have been used to find SLs, but they are thwarted by varius barriers such as high cost, off-target effects, and batch effects (Liu *et al.*, 2020). Hence, it is compelling to devise efficient computational methods to complement the downsides of the wet-lab screening techniques.

A spectrum of computational methods has been proposed for SL prediction. These methods can be categorized into three classes. The first class involves simulating *in silico* knockouts using metabolic network models. Folger *et al.* (2011) proposed to characterize SLs by modeling effects of the single- and double-knockouts of candidate genes in those networks. The second class, referred to as knowledge-oriented methods, is mostly conducted by feature engineering with domain-specific knowledge. To predict SL pairs, these methods employ network topology features such as graph centrality (Kranthi *et al.*, 2013), network flow (Zhang *et al.*, 2015), connectivity homology (Jacunski *et al.*, 2015) and features derived from genomic data including somatic copy number alteration (Jerby-Arnon *et al.*, 2014), short hairpin RNA profiles (Jerby-Arnon *et al.*, 2014), and gene expression profiles (Jerby-Arnon *et al.*, 2014). However, the two classes mentioned above rely heavily on the metabolic network models, domain knowledge and genomic data, without fully exploiting the valuable information of known SL pairs.

To exploit the exiting SL data, the third class of methods apply machine learning algorithms, where features are engineered based on both domain knowledge and heuristic functions. Paladugu *et al.* (2008) proposed to train a support vector machine (SVM) for SL prediction where the features were extracted from a protein–protein interaction (PPI) network. MetaSL (Wu *et al.*, 2014) integrated 17 features and weighted outputs of 10 classifiers to predict SLs. Aside from the traditional machine learning methods, graph representation learning approaches have been proposed, which mostly adopt an encoder–decoder paradigm. In this paradigm, an encoder tries to map the nodes into a low-dimensional embedding, whereas a decoder takes the embedding and utilizes it to reconstruct the node similarities in the original graph (Hamilton, 2020), thereby recovering missing links. This paradigm can be generalized to matrix factorization (MF)-based methods and graph neural network (GNN)-based methods. Those methods use distinct designs of encoders, but resemble each other in the choices of decoders (mostly taking the form of inner product predictor or its normalized variants). MF methods adopt a MF encoder. SL^2^MF (Liu *et al.*, 2020) proposed a MF encoder which decomposes the SL matrix, gene ontology (GO) similarity matrix, and PPI matrix to a low-dimensional latent space. GRSMF (Huang *et al.*, 2019) introduced a self-representative MF encoder which focuses on learning a representation matrix from known SL pairs and further integrates the functional similarities among genes derived from GO. Liany *et al.* (2020) adopted Collective Matrix Factorization (CMF) based methods to integrate data from heterogeneous sources to predict SLs.

The MF-based encoders are just shallow embedding methods, which simply optimize a unique embedding vector for each node, without sharing any parameter between nodes or leveraging node features (Hamilton, 2020). GNN, a state-of-the-art framework for deep learning on graphs, enhances the aforementioned methods by adopting a different embedding strategy. GNN defines a message-passing (MP) process on the original graph, i.e. at each iteration, each node aggregates all the embeddings from its local neighborhood as a message which is combined with its previous embedding to generate a new embedding. Based on GNN, Cai *et al.* (2020) adopted a novel regularization technique called dual dropout to address the sparsity of SL networks.

However, the existing GNN-based methods often regard each SL pair as an independent sample, and make no attempt to take their underlying biological mechanisms into account. However, some shared factors (such as biological processes, pathways, cellular components etc.) might latently invalidate the assumption of independency. For instance, poly (adenosine diphosphate-ribose) polymerase 1 (PARP1) and breast cancer 1 (BRCA1) are a famous SL gene pair, leading to the first clinically approved SL-based cancer drug, PARP inhibitor (Lord and Ashworth, 2017). PARP1 and BRCA1 are both key players in DNA repair process. Meanwhile, ATM and TP53 are another widely known SL pair (Kwok *et al.*, 2016), and ATM is also a key instrument in DNA repair process (Sanders *et al.*, 2020). Here, the DNA repair process might be the common mechanism underlying the two SL pairs.

A subset of existing methods [e.g. SVM, random forests (RFs), SL^2^MF and GRSMF] have injected some genomic and proteomic data to facilitate the SL prediction, and the results of these studies have underscored the significance of integrating additional information. Meanwhile, GNN-based methods can also encode such information as input features. However, these methods extracted features manually based on domain knowledge and some features might be left out. Therefore, to attain a more comprehensive set of features to improve the performance of SL prediction, we need a new method capable of automatic knowledge integration and feature extraction.

Knowledge graphs (KGs) are a type of multi-relational graph, where nodes and edges have different types. A KG is denoted by G=(V,E), where edges in set *E* are defined as triplets e=(h,τ,t) indicating a particular relationship τ∈T between two nodes (Hamilton, 2020). By incorporating a KG into a GNN, one can mitigate the aforementioned independency issue by directly introducing those latent factors as nodes in the graph. Lin *et al.* (2020) proposed an end-to-end knowledge GNN (KGNN) and achieved good performance in drug–drug interaction prediction.

Here, we propose a novel KGNN-based method for SL prediction, named KG4SL, which utilizes KG MP as a back-end. We approach the independency issue by injecting various factors including biological processes, diseases, compounds etc. that could be pertinent to SL, into our KG. Our model comprises three parts. In the first part, we derive a gene-specific subgraph from the original KG for each gene. In the second part, we conduct MP on the gene-specific subgraph, to automatically associate genes with factors that could be decisive in identifying an SL pair. In the third part, we define a decoder to reconstruct gene–gene similarity in a supervised fashion. To the best of our knowledge, this is the first framework to integrate KG with GNN for SL prediction. We compared our model with 10 state-of-the-art methods for SL prediction, and our model outperformed all the baselines in area under ROC curve (AUC), area under precision-recall curve (AUPR) and F1. Another contribution of our work is that we studied the impact of KG, which suggests that introducing a KG combined with MP process in GNN can significantly improve the performance of SL prediction.

## 2 Materials and methods

In this section, we first introduce the data and the problem of SL prediction. Then, we present the details of the proposed KG4SL model.

### 2.1 Data Collection

SynLethDB (http://synlethdb.sist.shanghaitech.edu.cn/v2/#/; Guo *et al.*, 2016) is a comprehensive database of synthetic lethal gene pairs. Its latest version includes a set of 36,402 human SL pairs, as well as a KG with 11 kinds of entities and 24 kinds of relationships as shown in Table 1. SynLethDB also includes negative SL pairs, i.e. Non-SL and synthetic rescue pairs. However, there are much less known negative SL pairs than known positive ones. To obtain a balance between the positive and negative samples, we randomly pick up unknown pairs as negative pairs so that there are equal numbers of positive and negative SL pairs. Hence, the final SL dataset contains 72 804 gene pairs between 10 004 genes.

**Table 1.** Details about the SL data and KG SynLethKG

SL data	No. of genes	10 004
	No. of interactions	72 804
	Density	0.14%
SynLethKG	No. of entity types	11
	No. of relationship types	24
	No. of nodes	54 012
	No. of edges	2 231 921

The KG, denoted as SynLethKG, includes 24 kinds of relationships between 11 entities. Among 24 kinds of relationships, 16 of them are related to genes directly, e.g. (gene, regulates, gene), (gene, interacts, gene) and (gene, co-varies, gene). And the other 8 relationships are associated with drug and compounds. Besides, 7 out of 11 kinds of entities are directly related to genes, i.e. pathway, cellular component, biological process, molecular function, disease, compound and anatomy. They are in the format of (gene, relationship, entity). These entities can be reached from genes in one hop, whereas the other three kinds of entities (pharmacologic class, side effect and symptom) can be reached from genes in two hops. After removing isolated nodes, the final graph of SynLethKG contains 54 012 nodes and 2 231 921 edges as shown in Table 1. Tables 2 and 3 show the details about the entities and relationships in SynLethKG. Users can access the SynLethKG through searching the names of the genes that they want to study in SynLethDB.

**Table 2.** Details about the entities in SynLethKG

Type	Sample size
Cellular component	1670
Gene	67 062
Biological process	12 703
Side effect	5726
Molecular function	3203
Pathway	2069
Disease	137
Compound	2595
Pharmacologic class	377
Anatomy	402
Symptom	453

**Table 3.** Details about the relationships in SynLethKG

Type	No. of edges	No. of source nodes	No. of target nodes
(Anatomy, downregulates, gene)	31	4	24
(Anatomy, expresses, gene)	617 175	241	23 881
(Anatomy, upregulates, gene)	26	5	22
(Compound, binds, gene)	16 323	1922	2306
(Compound, causes, side effect)	139 428	1079	5702
(Compound, downregulates, gene)	21 526	747	2847
(Compound, palliates, disease)	384	215	50
(Compound, resembles, compound)	6266	1034	1055
(Compound, treats, disease)	752	385	77
(Compound, upregulates, gene)	19 200	721	3205
(Disease, associates, gene)	24 328	135	6572
(Disease, downregulates, gene)	7616	44	5730
(Disease, localizes, anatomy)	3373	123	398
(Disease, presents, symptom)	3401	122	427
(Disease, resembles, disease)	404	100	98
(Disease, upregulates, gene)	7730	44	5614
(Gene, covaries, gene)	62 987	9174	9706
(Gene, interacts, gene)	148 379	9633	14 275
(Gene, participates, biological process)	619 712	16 608	12 703
(Gene, participates, cellular component)	97 652	11 916	1670
(Gene, participates, molecular function)	110 042	14 404	3203
(Gene, participates, pathway)	57 441	11 519	2069
(Gene, regulates, gene)	267 791	4649	7105
(Pharmacologic class, includes, compound)	1205	377	837

The 24 relationships in Table 3 describe the features of genes, drugs and compounds. These relationships are collected from Genbank, GO, Drugbank, DrugCental, PubMed, Bgee, String, LINCS L1000, SIDER4, STARGEO, Uberon and BioGRID. The specific number of each type of relationship and the number of associated nodes are also shown in Table 3. Besides, the types of the entities in SynLethKG and the number of each entity are shown in Table 2.

### 2.2 Problem statement

Formally, the SL data can be modeled as a matrix $S\in {\left(0,1\right)}^{n\times n}$, where *n* is the number of genes involved in the SL pairs. In this SL matrix *S*, an entry $s_{i,j}$ is 1 if there is an SL interaction between gene *e_i_* and gene *e_j_*, and 0 otherwise. Note that gene pairs with entry value 0 are unknown pairs, some of which could be potential SL pairs not yet discovered. The KG SynLethKG is denoted by $G=(V_{e},V_{r})$, which contains a set of entities *V_e_* and a set of relationships *V_r_*. Each edge in the KG is defined as a triplet T=(h,r,t), which shows a relationship of type *r* between head entity *h* and tail entity *t*, where $h,t\in V_{e}$ and $r\in V_{r}$.

Given the SL matrix *S* and the KG *G*, the problem we aim to solve is to predict the SL relationship between gene *e_i_* and gene *e_j_*. To achieve this goal, we propose a GNN-based model to learn a scoring function ${\hat{s}}_{i,j}=F(i,j|W,A,b)$ that estimates how likely gene *e_i_* and gene *e_j_* is an SL pair, where **W**, **A** and **b** denote the learnable parameters in function *F*.

### 2.3 Overview of KG4SL

The overall framework of KG4SL is laid out in Table 1. KG4SL utilizes a GNN to encode the gene features from KG for SL prediction in three steps. First, we derive a gene-specific weighted subgraph for each SL-related gene from the KG. Specifically, the weight of every edge is defined by a gene-specific relation scoring function to depict the importance of the relation for its target gene. Second, we design an aggregation layer to update the representation for a given gene by aggregating the representations of its neighbors in the gene-specific weighted subgraph. Third, we assign a score for each gene pair computed by the normalized inner product based on their learned representations. Next, we introduce these three steps in details.

#### 2.3.1 Gene-specific weighted subgraph

Given an SL-related gene, we first construct a weighted subgraph from the KG. Identifying relevant nodes and determining the edge weights are two key operations to construct the gene-specific weighted subgraph.

**Fig. 1.**
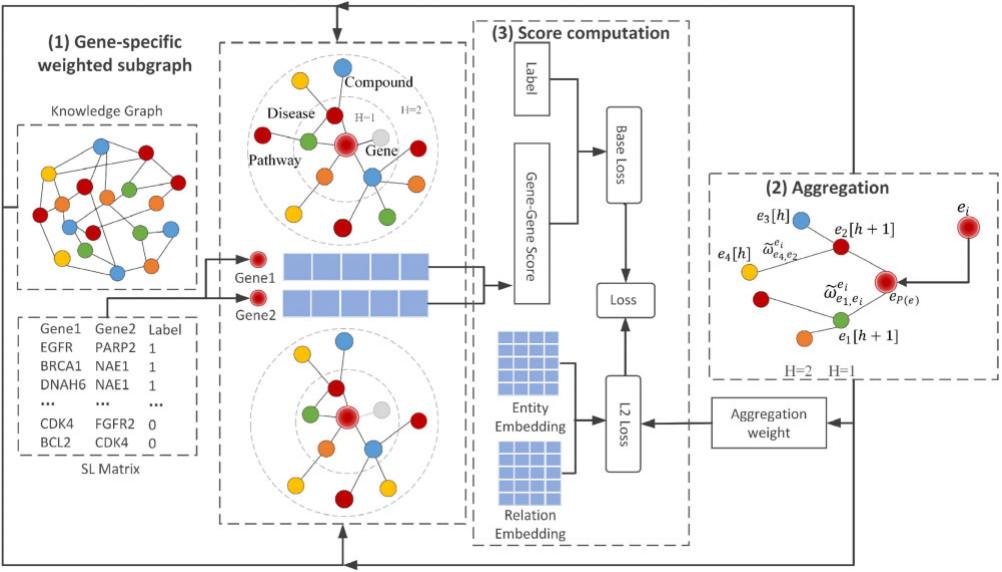
The framework of KG4SL. The workflow of KG4SL can be divided into three modules, including gene-specific weighted subgraph module, aggregation module and score computation module. (1) Gene-specific weighted subgraph: First, we construct a weighted subgraph from the KG. (2) Aggregation: Second, for each SL pair, we select the entities and relationships that are directly related to the nodes. Besides, we believe the biological information can flow between nodes through edges. Thus, we also aggregate the information of indirectly connected entities and relationships. Considering the problem of computing power, only two layers of entities and relationships are included. (3) Score computation: Third, the results of aggregation for two genes are used to compute their SL score through inner product. The loss function of KG4SL is composed of two kinds of losses, i.e. the base loss computed based on the truth label and the gene–gene score, and the *L2* loss computed using the entity embedding, relation embedding and aggregation weights

Assume that *e* is the central node/entity and *N*(*e*) is the set of neighbors of *e* (i.e. entities directly connected to *e*). In SynLethKG, the size of *N*(*e*) varies greatly among the entities. For example, network hubs may have thousands of relations, whereas some nodes are less studied and thus have a limited number of neighbors. In this work, we sample a fixed number of *k* neighbors for each entity to characterize its local structure and we repeat this process for *H* hops (H≥1). In particular, if a node has less than *k* neighbors, we sample duplicates, i.e. a neighbor may be sampled more than once. The set of sampled *k* neighbors is denoted as *P*(*e*). An example of 2-hop subgraph with neighbor sampling size *k *=* *4 in each hop can be seen in Figure 1.

In a gene-specific subgraph, we can assign different weights for edges to describe the importance of the relations. For an SL pair (*e_i_*, *e_j_*), the weight for an edge $r_{e,e\prime }$ in *e_i’_*s subgraph is computed by $\omega_{e,e\prime }^{e_{i}}=g(e_{j},r_{e,e\prime })$, where *e* is one of the entities in the subgraph of *e_i_*, and e′∈P(e). Besides, $e_{j}$ and $r_{e,e\prime }$ are the feature embeddings of gene *e_j_* and relation $r_{e,e\prime }$, respectively. *g* is an inner product function. Here, $\omega_{e,e\prime }^{e_{i}}$ characterizes the importance of relation $r_{e,e\prime }$ to gene *e_j_*.

#### 2.3.2 Aggregation of node representations

For any central entity *e* in the subgraph of gene *e_i_*, we aggregate the representations of all its picked neighbors to update its own representation. To show the topological neighborhood structure of entity *e* in the KG, we compute the weighted average combination of *e’*s neighborhood:

(1) $$e_{P(e)}=\sum_{e\prime \in P(e)}{\tilde{\omega }}_{e,e\prime }^{e_{i}}e\prime ,$$

where **e** is the representation of entity *e*, gene *e_i_* and gene *e_j_* are a pair in the SL matrix, and $\tilde{\omega }$ is the normalized gene-relation score by applying a softmax function:

(2) $${\tilde{\omega }}_{e,e`}^{e_{i}}=\frac{\;\text{exp}\;\left(\omega_{e,e`}^{e_{i}}\right)}{\sum_{\hat{e}`\in P(e)}\;\text{exp}\;\left(\omega_{e,\hat{e}`}^{e_{i}}\right)}.$$

After obtaining the picked neighbors’ representation $e_{P(e)}$ of a central entity in one hop, similar to (Wang *et al.*, 2019a), it integrates the entity representation **e** into a single vector to update **e**:

(3) $$e[h+1]=\phi \left(W\left(e[h]+e_{P(e)}\right)+b\right),$$

where **W** and **b** are the linear transformation weight and bias, respectively, and ϕ is an activation function such as *ReLU*. After aggregating neighbors’ information through *H* hops, the final feature representation of gene ${\hat{e}}_{i}$ is $e\left[H\right].\;\;{\hat{e}}_{j}$ is obtained in the same way.

#### 2.3.3 SL prediction score

Finally, by passing information from two subgraphs of KG, we attain the final representations ${\hat{e}}_{i}$ and ${\hat{e}}_{j}$ for the two genes in the SL matrix. The predicted interaction probability between gene *e_i_* and gene *e_j_* is calculated by ${\hat{s}}_{i,j}=\phi \left(f\left({\hat{e}}_{i},{\hat{e}}_{j}\right)\right)$, where *f* is the inner product function and ϕ is a sigmoid function, squashing the output to a range between 0 and 1. Furthermore, this link prediction can be viewed as a binary classification problem, by setting the threshold to 0.5. ${\hat{s}}_{i,j}$ is 1 or 0, which indicates whether an SL relation exists between a candidate pair of genes.

### 2.4 Overall loss and optimization

Two kinds of losses are designed for our model, including base loss and *L2* loss. The base loss *J* is computed through cross-entropy of the truth label and the predicted label for the edges, represented as follows.

(4) $$\begin{array}{cc} J & =\max({\hat{s}}_{i,j},0)-{\hat{s}}_{i,j}\times s_{i,j}+\text{log}(1+\text{exp}(-\left|{\hat{s}}_{i,j}\right|)), \end{array}$$

where ${\hat{s}}_{i,j}$ is the predicted label and $s_{i,j}$ is the truth label for the edge. We also add an *L2*-regularizer defined as:

(5) $$\begin{array}{c} ||\Gamma ||=\frac{\left||e||+||r||+||W|\right|}{2}, \end{array}$$

where ||·|| represents the *L2* norm for entity embedding, relation embedding and aggregation weights.

The final loss combines the two kinds of loss functions described above as follows:

(6) $${\min_{W,\;A,\;b}}_{}\ell ={\min_{W,\;A,\;b}}_{}\sum_{i,j}J+\alpha ||\Gamma ||,$$

where **A** is the trainable weighted matrix in which each element represents the gene-relation score and *L2* weight *α* is a balancing hyper-parameter. Here *α* was set to 0.0039. The first term corresponds to the part of GNN that learns the linear transformation weight **W**, gene-relation score weight **A** and bias **b** simultaneously. The second term added the *L2*-regularizer. Adam algorithm is used to minimize the final loss and the learning rate is set to 0.002. The framework of KG4SL is outlined in Algorithim 1.

**Algorithm 1 KG4SL**

**Input:** SL matrix *S*; KG $G(V_{e},V_{r})$; neighborhood field  *P*(*e*); hyper-parameters *α*,*d*, *k*, *h* and epoch;

**Output:**  ${\hat{s}}_{i,j}$

**1: Initialization:**
2: entity embedding matrix *W_e_*;
3: relation embedding matrix *W_r_*;
4: step ← 0;
**5: while** step < epoch **do**
**6:   for**  $(e_{i},e_{j})\in S$  **do**
7:    $GS_{i}{\left[h\right]}_{h=0}^{H}$ ← **G**ene-specific **S**ubgraph(*e_i_*);
8:    $GS_{j}{\left[h\right]}_{h=0}^{H}$ ← **G**ene-specific **S**ubgraph(*e_j_*);
**9:    for** *m* ∈[i,j]  **do**
10:     $e[0]\leftarrow GS_{m}[0]$;
**11:     for** *h* = 1,2,…,H **do**
**12:      for**  $e\in GS_{m}[h]$  **do**
13:       $e_{P(e)}[h-1]\leftarrow \sum_{e\prime \in P(e)}{\tilde{\omega }}_{e,e\prime }^{e_{m}}e\prime$;
14:       $e[h]\leftarrow \phi (W(e[h-1]+e_{P(e)}[h-1])+b)$;
**15:      end for**
**16:     end for**
17:     ${\hat{e}}_{m}\leftarrow e[H]$;
**18:    end for**
19:    Compute the predicted probability ${\hat{s}}_{i,j}\leftarrow \phi (f({\hat{e}}_{i},{\hat{e}}_{j}))$;
20:    Compute the loss ℓ;
**21:   end for**
22:   step ← step + 1;
**23:  end while**

## 3 Results

In this section, we first introduce the state-of-the-art baseline methods and their implementation details, and then we compare our model with the baselines, followed by an analysis of the influence of the KG. The KG4SL model was implemented with Python 3.6 and Tensorflow 1.15.0. We adopt AUC, AUPR and F1 as the evaluation metrics.

### 3.1 Performance evaluation

#### 3.1.1 Baselines

We compare KG4SL with the following baselines:

- **SL^2^MF** (Liu *et al.*, 2020) integrates gene similarities based on GO biological pathway annotations with SL matrix to predict SL pairs.
- **GRSMF** (Huang *et al.*, 2019) is a graph regularized self-representative MF model which also uses known SL pairs and GO-based gene similarities to predict SL pairs.
- **HOPE** (Ou *et al.*, 2016) is scalable to preserve high-order proximity of graphs and capable of capturing the asymmetric transitivity.
- **DeepWalk** (Perozzi *et al.*, 2014) is a graph embedding method which uses short random walks (RWs) to learn representations for nodes in graphs.
- **Node2Vec** (Grover and Leskovec, 2016) also learns feature representations for node in graphs but adds flexibility in exploring neighborhoods.
- **LINE** (Tang *et al.*, 2015) uses an effective edge-sampling method for model inference and preserves both the first- and second-order proximities by a fine-grained objective function.
- **Convolutional network (GCN)** (Kipf and Welling, 2016) is the most popular GNN architecture, which employs the symmetric-normalized aggregation as well as the self-loop update approach.
- **GraphSAGE** (Hamilton *et al.*, 2017) introduces the idea of generalized neighborhood aggregation.
- **GAT** (Veličković *et al.*, 2017) introduces the attention mechanisms to GNN.
- **DDGCN** (Cai *et al.*, 2020) is built to adapt to the sparsity of SL network, which includes a dual form drop out.

These baselines can be divided into three categories, MF-based baselines (SL^2^MF, GRSMF and HOPE), RW-based baselines (DeepWalk, Node2Vec and LINE) and GNN-based baselines (GraphSAGE, GAT, GCN and DDGCN). We evaluated DeepWalk, Node2Vec, LINE using CogDL (THUDM, 2020), GraphSage, GCN and GAT via graph convolution layer in DGL (Wang *et al.*, 2019b). Note that all the baselines do not utilize KG as inputs, so they are only trained on the SL interaction matrix. In addition to the SL interaction matrix, SL^2^MF and GRSMF also utilize GO semantic similarity matrices for SL prediction.

#### 3.1.2 Implementation details

All the baselines were evaluated at 5-fold cross validation which makes the best use of the available data. We viewed the input graph as an unweighted and undirected graph. For SL^2^MF and GRSMF, we utilized all the default parameters in the origin papers. For HOPE, the parameter beta was set to 0.02. For RW-based methods, the number of walks to start at each node was set to 5, the length of the RW start at each node was set to 10, the window size was set to 3. For node2vec, *p* and *q* were both set to 1, which respectively control how fast the walk explores and how fast the walk leaves the neighborhood of starting. For LINE, the alpha was set to 0.1 and other was set to 2. For DDGCN, we use the same setting of the origin paper. For GCN, GAT and GraphSage, we all used two convolution layers, and the dimensionality of the latent spaces in the first and second layers were chosen to be 5 and 16. The number of training epochs was decided via the early stopping strategy. KG4SL was evaluated using the ratio of training, validation and testing data as 8:1:1. In order to improve the stability of results, we randomly split dataset into 10 pieces and took one of them as the testing set. Early stopping strategy is used to control the number of training epochs.

#### 3.1.3 Comparison with baselines

On SynLethKG, KG4SL outperforms all baselines as shown in Table 4. When compared with the second best model GRSMF, KG4SL improves the performance on AUC, AUPR and F1 by 3.11%, 2.16% and 6.4%, respectively.

**Table 4.** Metrics of KG4SL against baselines in AUC, AUPR and F1

Categories	Methods	AUC	AUPR	F1
MF	SL^2^MF	0.7811 ± 0.0035	0.8635 ± 0.0021	0.7446 ± 0.0074
	GRSMF	0.9184 ± 0.0039	0.9362 ± 0.0023	0.8339 ± 0.0049
	HOPE	0.7776 ± 0.0005	0.7410 ± 0.0006	0.7089 ± 0.0010
RW	DeepWalk	0.8451 ± 0.0024	0.8600 ± 0.0013	0.7562 ± 0.0027
	node2vec	0.8362 ± 0.0010	0.8523 ± 0.0014	0.7503 ± 0.0031
	LINE	0.8233 ± 0.0028	0.8327 ± 0.0023	0.7380 ± 0.0056
GNN	GCN	0.8329 ± 0.0172	0.8727 ± 0.0110	0.8508 ± 0.0136
	GraphSAGE	0.8398 ± 0.0291	0.8775 ± 0.0188	0.8569 ± 0.0236
	GAT	0.7914 ± 0.0182	0.8462 ± 0.0103	0.8152 ± 0.0129
	DDGCN	0.8491 ± 0.0106	0.8998 ± 0.0056	0.8154 ± 0.0074
	KG4SL	**0.9470 **±** **0.0003	**0.9564 **±** **0.0005	**0.8877 **±** **0.0017

*Note*: The best results for each index are in bold.

In general, GNN-based models achieve better performance than shallow embedding methods like MF-based and RW-based models. This may because GNN-based models can learn from the similarity between SLs and enrich the embedding of genes for SL prediction. DDGCN represents the state-of-art model for SL prediction, and it achieves the best performance among the GNN-based baselines. MF-based method GRSMF is second only to KG4SL, which shows that the combination of GO gene similarity information and self-representation matrix decomposition is very effective for SL prediction. The performance of KG4SL is even higher, which shows that learning gene representations from the KG including GO information and other gene features can further improve SL prediction.

### 3.2 Model Analysis

#### 3.2.1 Parameter sensitivity

**Fig. 2.**
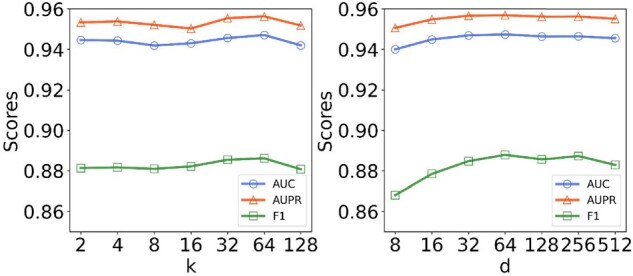
Parameter sensitivity analysis with varying *k* and *d* for KG4SL. Left: AUC, AUPR, and F1 at different neighbor sampling sizes *k*, ranging from 2 to 128. Right: AUC, AUPR and F1 at different node embedding dimensions *d*, ranging from 8 to 512

We present the sensitivity analysis for some key hyperparameters in our KG4SL, including the neighbor sampling size *k* and the dimension of entity embedding *d*, as shown in Figure 2.

First, we change the number of samples for neighbor *k* and observe the model performance. KG4SL achieves the best AUC, F1 and AUPR when the neighbor sampling size *k *=* *64. When sampling more neighbors with higher value of *k*, the information sampled may become redundant, and thus the model performance slightly decreases when *k* is 128. Next, we also investigate the influence of the dimension of embedding *d*. The KG4SL model already has a good performance when the dimension of embedding is 256. Too large dimension of embedding is a burden on memory and computation. Eventually, we set the neighbor sampling size as 64 and the dimension of embedding as 256 for our KG4SL model.

#### 3.2.2 Convergence analysis

**Fig. 3.**
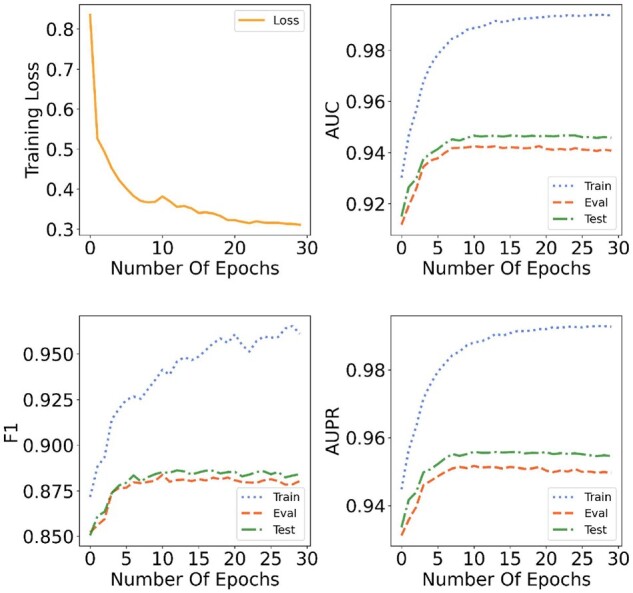
Convergence analysis of KG4SL. In the first subgraph, the orange line represents the change of training loss during the training process. In the other three subgraphs, the blue-dotted line represents the variation trend of three metrics (AUC, F1 and AUPR) during the training process. The red-dashed line displays the values of the above metrics for validation set as the increase of epochs. The green dash-dot line denotes the results for test set. At the first 10 epochs, the training loss drops rapidly and the three metrics for training set, validation set and test set increase with a uniform trend. Although at the 20th epoch, the training loss and three metrics begin to converge

With the above parameters set, we observe the convergence of the model. Figure 3 displays the change of loss and three metrics with the increase of epochs. The blue-dotted, red-dashed and green dash-dot lines represent the metrics of training data, validation data and testing data, respectively. The orange line shows the change of loss. We find that loss falls rapidly within the first 10 epochs and begins to converge gradually at the 20th epoch. Under the constraint of the *L2*-regularizer, loss converges to 0.3111 and the results of the three metrics in the training set, validation set and test set have the similar variation trend which shows that the proposed method can alleviate the problem of overfitting.

### 3.3 Impact of KG

While automatically integrating the KG into the node feature construction is crucial in our work, we wonder whether the KG is really important for the task of SL prediction. To investigate this problem, we test the SL prediction performance with and without the SynLethKG separately.

Table 5 shows the prediction performance of several machine learning models on SynLethKG, SL graph and the combination of both. KG-based methods intend to learn low-dimensional embeddings of entities and relations in SynLethKG automatically. We take TransE (Bordes *et al.*, 2013), a popular unsupervised KG embedding learning method as an example, which is trained based solely on SynLethKG. We also compare the contributions of a single SL graph, which is called SL-based method, whose performance is exactly that of GCN in Table 4. Then, a combination of the KG- and SL-based method, named ‘TransE + GCN’, is further tested. From the first three rows in Table 5, we can observe that additional information from the KG guides the model to achieve better performance than using KG or SL graph alone. TransE, trained on the KG only, reports an AUC score of 0.5870 and an AUPR of 0.6100, which are the lowest values among the three models. GCN, operating on the SL graph directly, leverages Xavier’s uniform distribution (Glorot and Bengio, 2010) as the initial node features, obtains AUC score of 0.8329 and AUPR score of 0.8727. The models that inspect the KG and SL graph together outperform either of the above. All the evidence supports that the KG information can help with SL prediction.

**Table 5.** Impact of KG analysis on AUC and AUPR

	Method	AUC	AUPR
KG-based	TransE	0.5870 ± 0.0086	0.6100 ± 0.0109
SL-based	GCN	0.8329 ± 0.0172	0.8727 ± 0.0110
KG + SL-based	TransE + GCN	0.9063 ± 0.0071	0.9138 ± 0.0056
	RF	0.8882 ± 0.0130	0.9218 ± 0.0098
	KG4SL	**0.9470 **±** **0.0003	**0.9564 **±** **0.0005

*Note:* The best results for each index are in bold.

After that, an ensemble learning method named RF (Breiman, 2001), which also uses the information extracted from KG and SL, is selected to be compared with ‘TransE + GCN’. The difference between RF and ‘TransE + GCN’ is that whether the features are extracted automatically. The features of RF should be carefully selected manually, whereas the gene embeddings are automatically extracted from TransE and fed into GCN to generate SL prediction results. Here, RF uses six features, namely, minTriangles, maxTriangles, minCoefficient, maxCoeffiecient, sp and sl. For each gene pair, minTriangles and maxTriangles reflect the max and min numbers of triangles that each gene forms. The minCoeffiecient and maxCoeffiecient reflect the min and max likelihood that the neighbors of these two genes are connected. sp is a Boolean value that represents whether the two genes are in the same community detected by the label propagation algorithm. sl means whether two genes are in the same community detected by Louvain algorithm (Blondel *et al.*, 2008). The results show that ‘TransE + GCN’ achieves slightly higher AUC and slightly lower AUPR than RF.

Comparing all these models with KG4SL which is an end-to-end model using the information extracted from KG and SL automatically, KG4SL yields the top AUC of 0.9470 and AUPR of 0.9564. This signifies the benefit of adding a suitable KG in SL prediction.

**Fig. 4.**
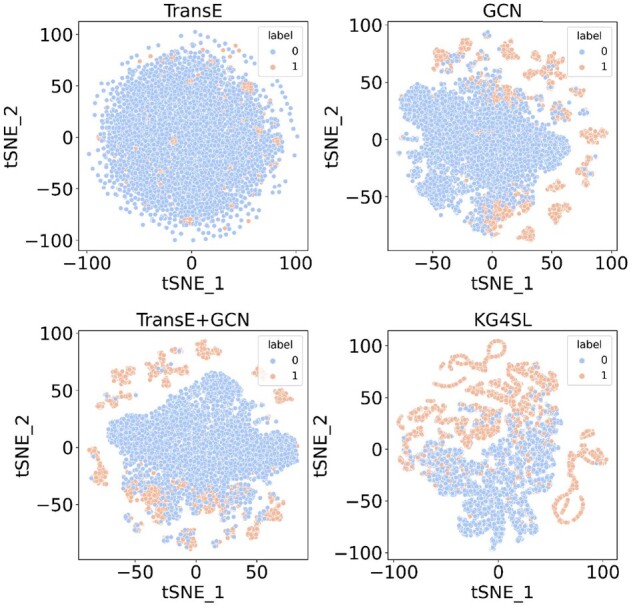
Visualization of SL interaction network. Link embeddings are generated by different models of gene–gene pairs in the SL matrix. Dot colors indicate whether there is an SL relationship between a pair of genes. TransE or GCN alone cannot distinguish the two types of links well as they only capture information from the KG or SL interaction, whereas KG4SL can distinguish the two link embeddings, demonstrating its ability to learn from both the SL network and the KG

Furthermore, to qualitatively interpret the above models’ learning abilities, we draw the link features extracted from these models. First, we fix the dimension of node features to 256. Next, the features for each node of an SL pair in test data are concatenated together, representing the link embedding between them. Then, the high-dimensional feature vectors are mapped into a 2D space by using visualization technique t-SNE (Van der Maaten and Hinton, 2008). As Figure 4 shows, orange dots denote there is an SL relation between a pair of test genes and blue dots are the opposite. We are curious whether the models can tell the differences between the two kinds of link types. Clearly, on one hand, TransE has the weakest distinguish capacity, as the SL label information is not taken into account. On the other hand, although both ‘TransE + GCN’ and KG4SL are integrated with the KG, KG4SL makes better use of this information, separating the two types of links more thoroughly.

## 4 Conclusion and discussions

In this article, we proposed a novel framework named KG4SL for predicting SL, which incorporates a knowledge graph (KG) into the GNN model. Many existing methods view each SL pair as an independent sample from a latent representation, but this assumption is an oversimplification in the biological context of cancer cells. KG can bring additional information such as biological processes which could be crucial for discovering new SL pairs. By injecting the KG, our model was capable of harnessing the aforementioned issue, without manual feature engineering. Extensive experiments have been conducted to examine the impact of KG. The results show that both KG and MP in GNN are essential for boosting the model performance. Therefore, KG4SL represents a breakthrough in applying supervised machine learning to SL prediction.

Our future work will focus on the following directions. First, we plan to further improve our model, using the strategy of contrastive learning, which is a self-supervised approach to learn graph representation. Secondly, the degrees of some nodes in a KG might be very large, while sampling a fixed-sized neighborhood may not fully capture the neighborhood topological structures of the nodes. To address this issue, it will be a highly desirable future work to contrast multiple receptive fields for a given node, to learn a more robust and enriched embedding. Moreover, inspired by the recently proposed KG attention network (Wang *et al.*, 2019c), we are interested in incorporating the attention mechanism into the KG MP. Considering there might be some promiscuous and uninformative neighbors for MP, the attention could play the role of a filter (Hamilton, 2020). By inspecting and visualizing the attention weights, interpretability analysis could be done to account for decisions made by GNN models. Thirdly, since our model outperforms most of the state-of-the-art models, it is desirable to utilize our model to discover novel SL pairs and collaborate with biologists to validate candidate SL pairs as drug targets. Last but not least, the Cancer Dependency Map (DepMap) project (Tsherniak *et al.*, 2017) aims to examine how perturbing a given target gene in a specific cancer type might affect the tumor growth, including cases when the best targets are SL partners of an altered gene. Das *et al.* (2019) predicted the SL pairs in the different cancer types which considered the tissue context. Wan *et al.* (2020) introduced the cell-line-specific gene expression information to help predict SL interaction, since most SL pairs remain cell-line specific. It is believed that such context-specific information can provide useful features for the SL prediction problem. Integrating the DepMap data into our KG4SL model, we can develop an AI system to facilitate the discovery of SL-based anticancer therapeutics. As many gene mutations cause cancer cells to inactivate, it is possible to kill the cancer cells by identifying the SL partners of these genes (O’Neil *et al.*, 2017). In the area of drug discovery or drug repurposing, using AI methods to narrow candidate drug targets set can speed up the research process.
